# Nonadditive Entropies and Complex Systems

**DOI:** 10.3390/e21050538

**Published:** 2019-05-27

**Authors:** Andrea Rapisarda, Stefan Thurner, Constantino Tsallis

**Affiliations:** 1 Dipartimento di Fisica e Astronomia and INFN Sezione di Catania, Via S. Sofia 64, 95123 Catania, Italy; 2 Complexity Science Hub Vienna, Josefstädterstrasse 39, A-1090 Vienna, Austria; 3 Section for the Science of Complex Systems, CeMSIIS, Medical University of Vienna, Spitalgasse 23, A-1090 Vienna, Austria; 4 Santa Fe Institute, 1399 Hyde Park Road, Santa Fe, NM 87501, USA; 5 Centro Brasileiro de Pesquisas Físicas and National Institute of Science and Technology of Complex Systems, Rua Dr. Xavier Sigaud 150, 22290-180 Rio de Janeiro, Brazil

**Keywords:** complex systems, nonadditive entropies, nonextensive statistical mechanics, beyond Boltzmann–Gibbs–Shannon

An entropic functional *S* is said *additive* if it satisfies, for any two probabilistically independent systems *A* and *B*, that S(A+B)=S(A)+S(B). If not, it is said *nonadditive*. In the literature, since the pioneering works of Boltzmann (1872, 1877) [1,2], Gibbs (1902, 1948) [3,4], von Neumann (1927) [5], and Shannon (1948, 1949) [6,7], dozens and dozens of entropic functionals have been defined along the years. Only two of them are additive, namely the Boltzmann–Gibbs–von Neumann–Shannon one (hereafter referred to as $S_{BG}$, where BG stands for Boltzmann–Gibbs), and the Rényi one $S_{q}^{R}$ (1961, 1970) [8,9]. All others are generically nonadditive. Let us mention some of them, namely $S_{q,r}^{SM}$ (Sharma–Mittal 1975) [10], $S_{q}$(Tsallis 1988) [11], $S_{q}^{A}$ (Abe 1997) [12], $S_{q,q^{\prime }}^{BR}$ (Borges–Roditi 1998) [13], $S_{q}^{LV}$ (Landsberg–Vedral 1998) [14], $S_{b}^{C}$ (Curado 1999) [15], $S_{\eta }^{AP}$ (Anteneodo–Plastino 1999) [16], $S_{\kappa }^{K}$ (Kaniadakis 2001) [17], $S_{f(\beta )}$ (Tsallis–Souza 2003) [18], $S_{q,q^{\prime }}$ (Schwammle–Tsallis 2007) [19], $S_{\delta }$ (Shafee 2007 [20], Tsallis 2009 [21], Ubriaco 2009 [22]), $S_{a_{n}}^{T}$ (Tempesta 2011) [23], $S_{c,d}^{HT}$ (Hanel–Thurner 2011 [24]), $S_{q,\delta }$ (Tsallis–Cirto 2013 [25]), $S_{a,b,r}$ (Curado–Tempesta–Tsallis 2016) [26], $S_{Z}^{T}$ (Tempesta 2016) [27], $S_{G}^{JPPT}$ (Jensen–Pazuki–Pruessner–Tempesta 2018) [28], among various others. They have many connections and predecessors in areas such as cybernetics, information theory, engineering, communication theory, ecology, and information geometry. All of these entropies recover the celebrated entropy $S_{BG}$ as a particular case, with the unique exception of $S_{b}^{C}$.

As it is well known, the entropy $S_{BG}$ and its associated statistical mechanics enable the correct calculation of a large variety of thermostatistical properties at or near thermal equilibrium of uncountable so-called simple systems. However, when it comes to wide classes of so-called complex systems the BG theory fails. Due to this fact, many attempts have emerged using either the Rényi entropy or some of the nonadditive ones, most frequently $S_{q}$, for a variety of applications in natural, artificial, and social systems.

In the present Special Issue, several approaches have been advanced along those lines. Following the order of appearance, Rodriguez et al. [29] have focused on a classical *d*-dimensional many-body Hamiltonian with long-range interactions, which numerically appears to exhibit *q*-Gaussian distributions of velocities, *q*-exponential distribution of energies, and vanishing maximal Lyapunov exponent in the infinitely-sized limit. Curado et al. [30] focus on a close relationship between the entropy $S_{q}$ and systems exhibiting power-law frequency of events and behaving similarly to self-organised criticality, like earthquakes, avalanches, and forest fires. Viallon–Galinier et al. [31] experimentally study the rheology of dense granular systems, exhibiting *q*-Gaussian distributions of displacements with an anomaly in the evolution of the index *q* directly related to percolating shear bands. Hanel et al. [32] focus, in the context of statistical inference, on a maximum configuration predictor for driven systems with arbitrary driving, and also discuss the associated Legendre structure. Obregon et al. [33] focus on quantum superstatistics and the critical behaviour of generalised ideal Bose gases. Ibrahim et al. [34] perform an analytic study of complex fractional entropy $S_{q}$ and apply it to complex neural networks. Zhao et al. [35] discuss the hedging for the regime-switching price model based on nonextensive statistical mechanics. Cetin et al. [36] verify a generalised Pesin-like identity and scaling relations at the chaos threshold of the Rössler ordinary differential equations in all three of its continuous variables. In a second contribution by Zhao et al. [37] a non-Gaussian stochastic process based on nonextensive statistical mechanics is employed, which can satisfactorily describe characteristics of long-run dependence of asset prices, and, by using the martingale method, closed form solutions are obtained for geometric average Asian options. Finally, Jensen et al. [38] exhibit how the phase space geometry leads, via group theory, to quite general entropy functionals where the composability plays a relevant role.

The ensemble of these contributions illustrates the power of nonadditive entropies in the realm of complex systems and outlines various interesting perspectives for the future.
